# Proanthocyanidin-Conjugated NIR-ΙΙ Nano-Prodrugs for Reversing Drug Resistance in Photothermal Therapy

**DOI:** 10.3390/molecules30112334

**Published:** 2025-05-27

**Authors:** Lan Cui, Weishuang Lou, Xin Wei, Mengdi Li, Mengyao Sun, Siyue Wang, Shuoye Yang, Lu Zhang, Guangzhou Zhou, Peng Li, Lingbo Qu

**Affiliations:** 1 College of Biological Engineering, Henan University of Technology, Zhengzhou 450001, China; cuilanmm@haut.edu.cn (L.C.); 13598611016@163.com (W.L.); 18236139462@163.com (X.W.); 17513121786@163.com (M.L.); 17737879593@163.com (M.S.); w200408170546@163.com (S.W.); yangshuoyecpu@163.com (S.Y.); zhanglu@haut.edu.cn (L.Z.); 2 Institute for Complexity Science, Henan University of Technology, Zhengzhou 450001, China; 3 College of Chemistry, Zhengzhou University, Zhengzhou 450001, China; qulingbo@zzu.edu.cn

**Keywords:** proanthocyanidin, NIR-ΙΙ photothermal therapy, prodrug, drug resistance

## Abstract

Targeting and multidrug resistance are the significant problems of current antitumor drugs, and these problems become the key factors in the design of nanomedicine. Herein, Au NRs and OPC-Au NPs were prepared via the hydroquinone seedless growth method and proanthocyanidin (OPC) one-pot method, and then pH-GSH-near-infrared ΙΙ (NIR-ΙΙ)-responsive nano-prodrugs Au/DOX-ss LNRs and OPC-Au/DOX-ss LNPs were designed by the encapsulation of doxorubicin prodrug DOX-ss with Au-S affinity and thermal-sensitive liposomes. Interestingly, OPC endowed OPC-Au NPs with reducibility and excellent performance in terms of particle size, zeta potential, encapsulation rate, and drug loading rate. In particular, the photothermal efficiencies of OPC-Au/DOX-ss LNPs increased to 59.22% under the 1064 nm NIR-ΙΙ irradiation. Compared with free DOX-ss and Lipid DOX-ss, the IC50 of OPC-Au/DOX-ss LNPs was decreased by 91.68% and 97.60%, respectively. Furthermore, the expression of P-gp in MCF-7/ADR was significantly inhibited (decreased by 65%). The potential of proanthocyanidin remodels the pH-GSH-NIR-ΙΙ responsiveness and drug resistance of OPC-Au/DOX-ss LNPs for breast cancer treatment in NIR-ΙΙ photodynamic/photothermal therapy.

## 1. Introduction

Chemotherapeutic drugs are always associated with significant side effects, such as cardiotoxicity, myelosuppression, and multidrug resistance [1,2]. Although liposome encapsulation has been reported to reduce drug toxicity through transmembrane transport, ensure the integrity and bioactivity of drug delivery, and improve pharmacokinetic effects in vivo [3,4]. It is difficult for hydrophilic drugs to diffuse from the liposome membrane because of its dense gelatinous crystalline state of the liposome membrane at normal body temperature [5]. Nowadays, thermal-sensitive liposomes have been designed to be controllable fluids at local high temperatures to promote drug release by enhancing molecular motion [6,7]. However, how to minimize drug toxicity and improve drug targeting sensitivity is still the key to the design of thermal-sensitive liposomes.

Gold nanoparticles have attracted great interest in photothermal therapy because of their special surface plasmon resonance effect, excellent photothermal stability, and robust absorption in the near-infrared region [8,9]. However, the synthesis of gold nanoparticles involving the template, photochemical, electrochemical, seed-mediated, one-step chemical oxidation, and seedless growth methods has disadvantages of complex operation, long reaction time, low toxicity of templates, and low yield of gold nanoparticles [10]. It is urgent to develop a green and pollution-free method for preparing near-infrared ΙΙ (NIR-ΙΙ) gold nanomaterials.

Proanthocyanidins (OPCs) are naturally occurring polyphenols with metal chelation, reduction, hydrogen bonding, and free radical scavenging properties [11,12,13]. Moreover, OPC can also mitigate oxidative stress damage, prevent tumor progression, and promote apoptosis by down-regulating molecular content, scavenging free radicals, and inhibiting MAPK/AKT signaling pathways [14,15,16,17]. Although gold nanoparticles could be designed by hydrothermal and one-pot methods based on the reducibility and stability of polyphenols [18], near-infrared ΙΙ photothermal properties could not be achieved. Importantly, the local high temperature during photothermal therapy tends to induce high expression of heat shock proteins, thus affecting the efficiency of chemotherapy [19,20,21,22,23,24]. The focus of photothermal reagent design is how to improve the photothermal conversion efficiency under mild and NIR-ΙΙ light.

**Scheme 1:**
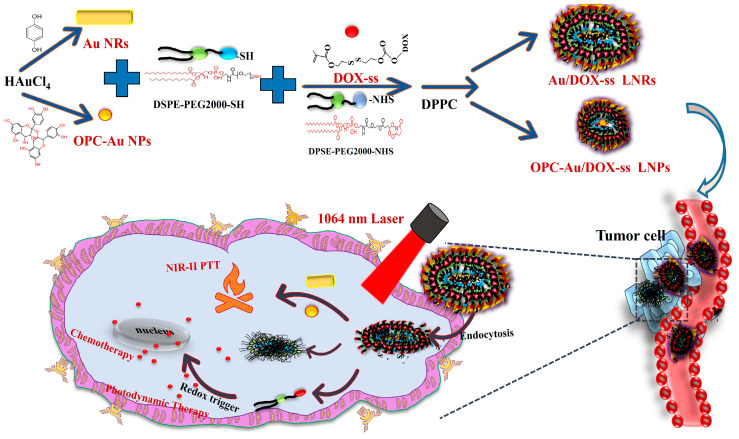
Schematic diagram of green preparation and remodeling targeted drug resistance of polyphenol-conjugated gold nano-prodrugs by synergistic pH-GSH-NIR-ΙΙ phototherapy.

In this work, polyphenol–gold NIR-ΙΙ nano-prodrugs Au/DOX-ss LNRs and OPC-Au/DOX-ss LNPs were constructed and connected by Au-S affinity and thermal-sensitive liposome encapsulation through the previously obtained doxorubicin prodrug DOX-ss and liposome synthesis methods. The effect of polyphenol structure of hydroquinone and proanthocyanidin on the drug-resistant sensitivity was evaluated to exploit the particle sizes, potentials, UV absorption peaks, drug loading rates, photothermal conversion efficiency, pH-GSH-NIR-ΙΙ-responsive drug release, biocompatibility, and targeted cytotoxicity to breast cancer-resistant cells (MCF-7/ADR). The aim of this study is to promote the clinical development of pH-GSH-NIR-ΙΙ photodynamic/photothermal combination therapy for breast cancer resistance (Scheme 1).

## 2. Results

### 2.1. Effects of Polyphenol Structure on Gold Nanoparticles

Herein, Au NRs were synthesized by the seedless growth method using hydroquinone as a reducing agent, and the homogeneous and dispersed OPC-Au NPs were synthesized by one-pot green synthesis method using proanthocyanidins as a reducing, stabilizing, and modifying agent, respectively.

**Figure 1:**
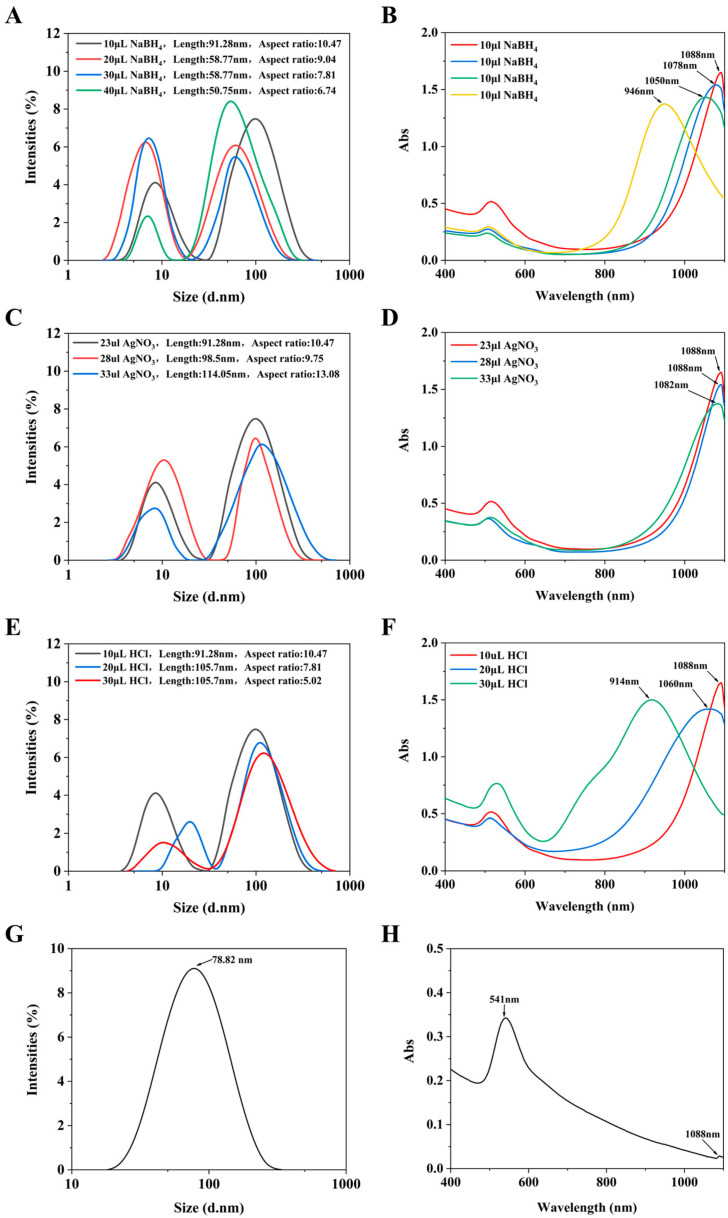
Effect of polyphenol structure on the growth of gold nanoparticles. (**A**,**B**) Effect of NaBH_4_ on the particle size and UV absorption of Au NRs. (**C**,**D**) Effect of AgNO_3_ on the particle size and UV absorption of Au NRs. (**E**,**F**) Effect of HCl on the particle size and UV absorption of Au NRs. (**G**,**H**) Particle size and UV absorption analysis of OPC-Au NPs.

As shown in Figure 1A,B and Figure S1A, the particle sizes of Au NRs decreased from the initial 91.3 nm to 50.8 nm, but the percentage of gold nanoparticles with smaller particle sizes gradually rose as the dose of NaBH_4_ (HCl: 10 μL, AgNO_3_: 23 μL) increased. Moreover, the potential of Au NRs decreased from 31.4 mV to 16.3 mV, but the stability worsened. Meanwhile, the absorption peaks of Au NRs were gradually blue shifted from 1088 nm to 946 nm. This might be ascribed that NaBH_4_ is a powerful reducing agent to promote the bursts into nucleation by rapid random attachment and intra-particle ripening through LaMer action [21,22]. However, the generation of gold nanoparticles of all sizes could be triggered due to the stochastic nature of the nucleation process, and smaller Au NRs would be rapidly induced through the high concentration of NaBH_4_, thus affecting the homogeneity of Au NRs [22,23].

Figure 1C,D and Figure S1B revealed that the particle sizes of Au NRs gradually rose from 91.3 nm to 114.1 nm, and their potential also gradually reduced and became unstable as the dose of AgNO_3_ (NaBH_4_: 10 μL, HCl: 10 μL) increases. However, the UV absorption of Au NRs was almost unchanged. This might be attributed to the fact that the surfactant and AgNO_3_ preferentially bind to the crystal faces (100) or (110) of gold during the crystal growth process, thus affecting the accumulation of gold atoms, inhibiting the nucleation of Au (0) on the (100) face and enabling the preferential growth of the stable face (111) to generate gold rods. AgNO_3_ plays a dominant role in adjusting the aspect ratios of gold nanorods; the excess of Ag^+^ only affects the transverse deposition of gold nanorods, but the ionic strength effect of Ag^+^ was weak on the transverse resonance absorption peaks of gold nanorods [23,24,25].

As shown in Figure 1E,F and Figure S1C, the particle sizes of Au NRs gradually increased from the initial 91.3 nm to 105.7 nm with the increase in HCl dose (NaBH_4_: 10 μL, AgNO_3_: 23 μL). Moreover, their potential also slightly declined, while the absorption peaks were gradually blue shifted from 1088 nm to 914 nm. This might be attributed to the fact that HCl could inhibit the reduction of hydroquinone and NaBH_4_, prolong the reduction time, and effectively hinder the growth of Au NRs [26,27].

According to the above analysis, Au NRs that were synthesized with hydroquinone as a reducing agent were optimized and exhibited an aspect ratio of 7.95 (long diameter: 91.3 nm, short diameter: 11.6 nm), and the zeta potentials were always positive (31.4 mV).

As shown in Figure 1G,H and Figure S1D, OPC-Au NPs exhibited excellent performance. The particle size and zeta potential were 78.8 nm and −23.5 mV, respectively. Furthermore, the characteristic absorption peak at 541 nm presented an asymmetric narrow shape, and a weak absorption peak appeared at 1088 nm. The proanthocyanidin-encapsulated gold nanocomposites were formed due to the hydroxyl structures and their reducing properties of proanthocyanidins based on the monomeric units flavan-3-ol (+) catechin and (−) epicatechin. The extensive hydroxyl groups ultimately prompt the reduction of gold ions into gold atoms and interact with the gold surface to form the shells of gold nanoparticles [28]. It has been found that small nanoparticles are easy to diffuse into deeper parts of tumor tissues and enter into tumor cells through cytosis [29]. In addition, positively charged carriers are easily captured by macrophages during blood circulation and cause inflammation and tissue damage upon binding with immune proteins. However, negatively charged or uncharged nanomedicines exhibit excellent characteristics to achieve long circulation in vivo [30,31,32]. Thus, OPC-Au NPs that were synthesized with procyanidins as a reducing agent exhibited a particle size of 78.8 nm, and the surface was charged negatively, which could avoid being phagocytosed by proteins in the bloodstream, prolong circulation time in vivo, and be more prone to target tumor sites during blood circulation.

In addition, NaBH_4_ is prone to causing skin and eye irritation, whereas OPCs are widely found in vegetables and fruits and exhibit good biocompatibility and safety without further addition of reducing agents, modifiers, or surfactants to form nanostructures with homogeneous size and shape [33,34].

Therefore, OPC-Au NPs using proanthocyanidins as reducing and stabilizing agents through the one-pot method were more conducive in the subsequent study of drug-carrying nano-systems.

### 2.2. Chemical Characteristics of Polyphenol-Conjugated Gold Nano-Prodrugs

**Figure 2:**
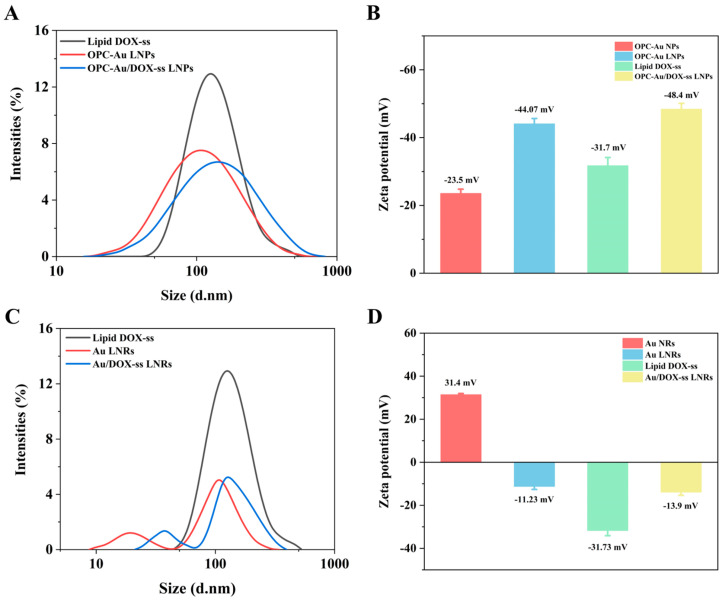
Chemical analysis of polyphenol–gold nano-prodrugs. (**A**,**B**) Particle size and potential of OPC-Au/DOX-ss LNPs. (**C**,**D**) Particle size and potential of Au/DOX-ss LNRs.

Au NRs and OPC-Au NPs were successfully encapsulated by liposomes and polyphenol-conjugated gold nano-prodrugs. OPC-Au/DOX-ss LNPs and Au/DOX-ss LNRs were synthesized with a higher encapsulation rate and drug loading rate (Figure 2). Figure 2A,B show the particle size and zeta potential of OPC-Au/DOX-ss LNPs. The particle sizes increased significantly from the initial 78.8 nm to 105.7 nm, and the potential changed from −23.50 mV to −44.07 mV after liposome encapsulation. After further encapsulation of Lipid DOX-ss, the particle size and zeta potential further increased to 141.8 nm and −48.40 mV, indicating the successful synthesis of polyphenol-conjugated gold nano-prodrugs OPC-Au/DOX-ss LNPs. Furthermore, the negative charge was favorable for the drug-carrying nanoparticles to enter the tumor cells. In addition, the encapsulation rate and drug loading rate of OPC-Au/DOX-ss LNPs were 84.80% and 21.76%, respectively, which exhibited a good drug loading performance.

As illustrated in Figure 2C,D, the long and short diameters of Au NRs increased to 105.7 nm and 18.2 nm, respectively, and their potential changed from 31.4 mV to −11.23 mV after liposome encapsulation. After further encapsulation of Lipid DOX-ss, the particle size was further increased to 122.4 nm, and its potential was −13.90 mV, demonstrating that Au/DOX-ss LNRs were successfully synthesized. The encapsulation rate and drug loading rate of Au/DOX-ss LNRs were 94.80% and 26.67%, which exhibited good drug loading performance.

The FT-IR spectra of OPC-Au/DOX-ss LNPs and Au/DOX-ss LNRs are shown in Figure S2A,B. The broad and intense adsorption peak at 3392 cm^−1^ corresponds to the stretching vibration peak of the hydroxyl on the aromatic ring of OPC. The peaks at 1614 cm^−1^, 1523 cm^−1^, and 1446 cm^−1^ were associated with the backbone vibration of the aromatic ring on the benzopyran ring. The peak shifts to 3430 cm^−1^ with a narrower peak shape compared with OPC, which illustrates the redox reaction between the phenolic hydroxyl of proanthocyanidins and Au^3+^. The skeletal vibrational peaks of the aromatic ring on the benzopyran ring of OPC-Au are shifted to 1635 cm^−1^, 1523 cm^−1^, and 1401 cm^−1^, which may be ascribed to the redox reaction of phenolic hydroxyl. The position and absorption peaks on the benzene ring framework were changed due to the enhanced electron-donating ability of the oxygen anion to the benzene ring [34,35]. These results suggested the successful synthesis of OPC-Au NPs.

In addition, the characteristic absorption peaks at 805 and 1220 cm^−1^ are associated with DOX, the C-OH and C-C characteristic peaks of DOX at 1285 and 1412 cm^−1^, the bi-carbonyl stretching vibration peak of DOX at 1614 cm^−1^ [36,37] and the appearance of S-S stretching vibration peak at 539 cm^−1^ proved that disulfide bond was successfully connected to DOX. A total of 1739 cm^−1^ was the carbonyl stretching vibration peak of DSPE-PEG 2000 [38], 2918 cm^−1^ and 2854 cm^−1^ represent the CH alkyl peaks of DSPE-PEG 2000 [36]. These results indicated that the liposomes successfully encapsulated DOX-ss. The characteristic absorption peak of the Au-S bond at 765 cm^−1^ [39] indicated that the OPC-Au NPs and Au NRs were attached to Lipid DOX-ss. OPC-Au/DOX-ss LNPs and Au/DOX-ss LNRs were successfully synthesized.

### 2.3. Effects of Polyphenol Structure on Photothermal Conversion and Drug Release

**Figure 3:**
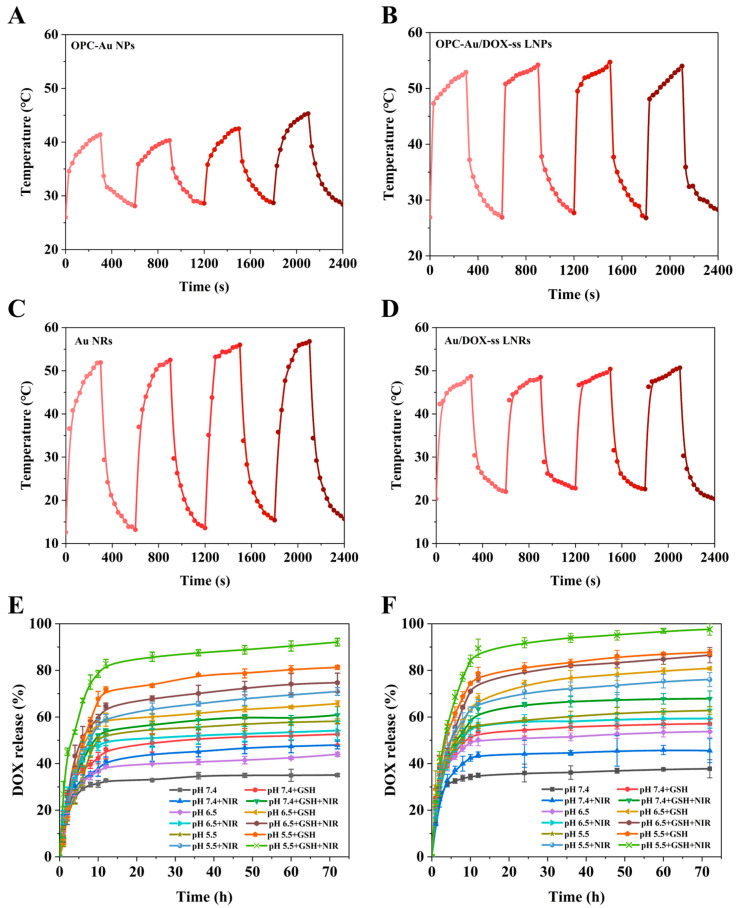
Photothermal conversion of polyphenol–gold nano-prodrugs. (**A**,**B**) Photothermal conversion curves of OPC-Au NPs and OPC-Au/DOX-ss LNPs. (**C**,**D**) Photothermal conversion curves of Au NRs and Au/DOX-ss LNRs. (**E**) Drug release of OPC-Au/DOX-ss LNPs. (**F**) Drug release of Au/DOX-ss LNRs.

As shown in Figure 3A–D, OPC-Au NPs, OPC-Au/DOX-ss LNPs, Au NRs, and Au/DOX-ss LNRs exhibited good photostability. The photothermal conversion efficiencies of OPC-Au NPs and OPC-Au/DOX-ss LNPs under 1064 nm NIR-ΙΙ light irradiation were 47.10% and 59.22%, respectively. Meanwhile, the photothermal conversion efficiencies of Au NRs and Au/DOX-ss LNRs under 1064 nm NIR-ΙΙ light irradiation were 66.53% and 55.34%, respectively. The above data indicated that NIR-II region could produce higher photothermal effects in biological tissues through a deeper penetration depth compared to the NIR-I region. Interestingly, the photothermal conversion efficiencies were enhanced through OPC conjugation on the surface of OPC-Au/DOX-ss LNPs.

As shown in Figure 3E,F, both OPC-Au/DOX-ss LNPs and Au/DOX-ss LNRs showed significant pH-GSH-NIR-ΙΙ responsiveness. The release rates of OPC-Au/DOX-ss LNPs at pH 5.5 (58.18%), pH 5.5 + GSH (81.28%), pH 5.5 + NIR-ΙΙ (70.91%), and pH 5.5 + GSH + NIR-ΙΙ (92.09%) were 1.65, 2.31, 2.02, and 2.63 times higher than that of pH 7.4 (35.08%) conditions, respectively. The release rates of Au/DOX-ss LNRs at pH 5.5 (62.74%), pH 5.5 + GSH (87.73%), pH 5.5 + NIR-ΙΙ (76.03%), and pH 5.5 + GSH + NIR-ΙΙ (97.57%) were 1.66, 2.32, 2.01, and 2.58 times higher than that of pH 7.4 (37.78%) conditions, respectively. This might be ascribed that Au-S breaks more readily under low pH conditions, and the drug release rate shows a significant pH responsiveness [40]. The photosensitizer warms up after irradiation by NIR light, which leads to a change in the membrane permeability of the heat-sensitive liposome and an increase in drug release, thus further enabling the drug delivery system to accelerate drug release under NIR light irradiation [41]. Meanwhile, the introduced disulfide bond has a good GSH response, which makes the drug-carrying nanoparticles have better drug release performance and present GSH responsiveness [42]. The pH-GSH-NIR responsiveness of the OPC-Au/DOX-ss LNPs and Au/DOX-ss LNRs system facilitated the drug release in tumor tissues and reduced toxic side effects on normal tissues.

### 2.4. Targeting Selectivity to Multidrug-Resistant Breast Cancer Cells

**Figure 4:**
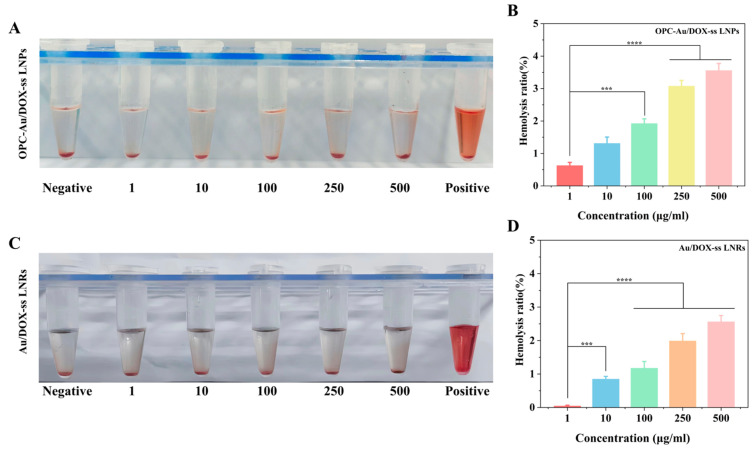
Hemolytic analysis of polyphenol-conjugated gold nano-prodrugs. (**A**) Hemolytic analysis of OPC-Au/DOX-ss LNPs. (**B**) Hemolysis ratio of OPC-Au/DOX-ss LNPs. (**C**) Hemolytic analysis of Au/DOX-ss LNRs. (**D**) Hemolysis ratio of Au/DOX-ss LNRs. *** and **** mean *p* < 0.001 and *p* < 0.0001.

**Figure 5:**
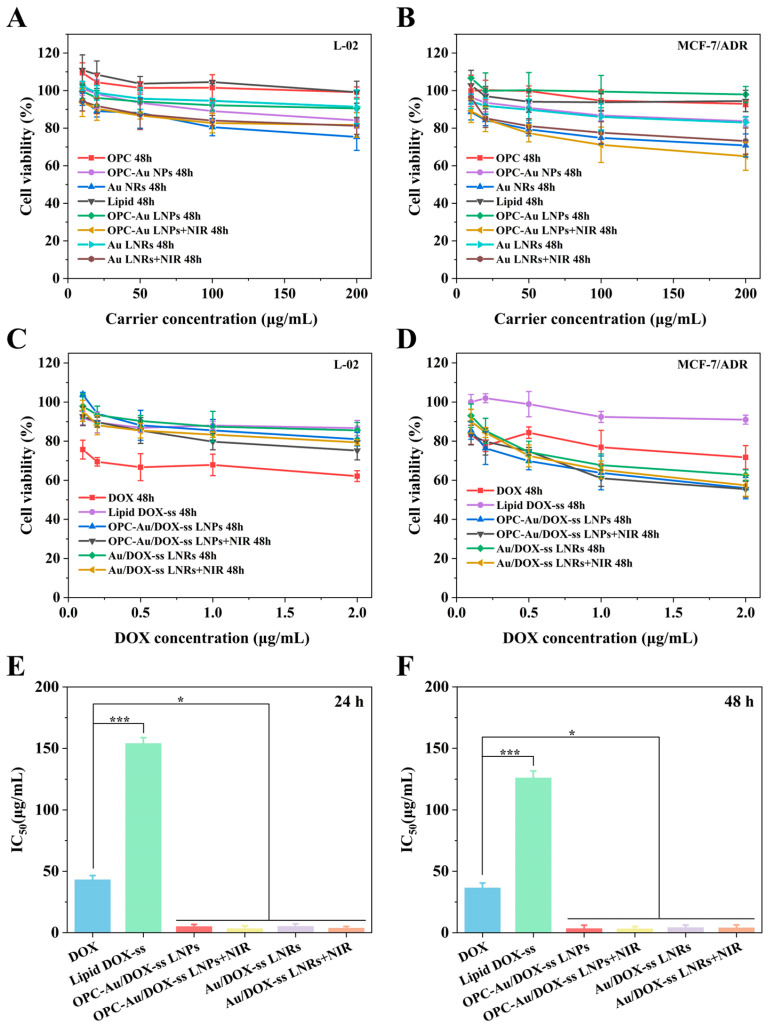
Selectivity analysis of polyphenol-conjugated gold nano-prodrugs in L-02 and MCF-7/ADR cells after 48 h of incubation. (**A**,**B**) Cell survival of empty carrier. (**C**,**D**) Cell survival of polyphenol–gold nano-prodrugs. (**E**,**F**) IC_50_ values of polyphenol–gold nano-prodrugs in MCF-7/ADR cells. * and *** mean *p* < 0.05 and *p* < 0.001.

As shown in Figure 4, the hemolysis rates of OPC-Au/DOX-ss LNPs and Au/DOX-ss LNRs were less than 5% in the range of the tested concentrations, indicating that both of them presented good biocompatibility. Moreover, the cell viability of empty carriers of OPC, OPC-Au NPs, Au NRs, Lipid and OPC-Au LNPs, and Au LNRs was above 80% after 24 h and 48 h incubation in human normal hepatocytes (L-02) and multidrug-resistant breast cancer cells (MCF-7/ADR), showing good biosafety. In addition, the combination of NIR irradiation may cause certain cytotoxicity in Au NRs and Au LNRs groups at a concentration of 200 μg/mL in human normal hepatocytes (L-02), while the OPC conjugation on the surface of OPC-Au LNPs could shield the cytotoxicity of Au (Figure 5A,B and Figure S3A,B). Notably, after NIR irradiation, the cell viability of OPC-Au LNPs + NIR and Au LNRs + NIR groups after 48 h was decreased by 9.8%, 11.05% (L-02 cell), and 33.57% and 11.92% (MCF-7/ADR cell) compared with that in the absence of NIR-ΙΙ light irradiation, respectively, indicating that OPC-Au LNPs had a better targeting selectivity to multidrug-resistant breast cancer cells due to the OPC conjugation on the surface and the combined PTT effect.

Figure 5C and Figure S3C showed that DOX was more toxic to L-02 cells with a cell survival rate of 62.10% after 48 h of incubation (DOX: 2 μg/mL). Interestingly, the toxicity was significantly reduced, and the cell survival rate of Lipid DOX-ss was considerably increased to 86.68% after the prodrug design and liposome encapsulation. The cell survival rates of OPC-Au/DOX-ss LNPs and Au/DOX-ss LNRs were both slightly declined compared with Lipid DOX-ss, but also above 80%, which were less toxic to normal L-02 cells, and exhibited better biosafety.

However, polyphenol-conjugated gold nano-prodrugs significantly improved the sensitivity of MCF-7/ADR cells, especially the sensitivity was significantly enhanced in the OPC-Au/DOX-ss LNPs group than the DOX Au/DOX-ss LNRs group (Figure 5D and Figure S3D). As shown in Figure 5D, the cell viability of the DOX group was 71.71% after 48 h incubation in MCF-7/ADR cells. The cell survival rate of the Lipid DOX-ss group was increased to 90.98% due to the good biocompatibility of the liposome. Whereas the survival rates of OPC-Au/DOX-ss LNPs and Au/DOX-ss LNRs were declined to 55.96% and 62.67%. The cytotoxicity of Au NRs is comparable to that of DOX-loaded Au/DOX-ss LNPs due to the fact that the cytotoxicity of Au NRs was slightly greater than that of OPC-Au, which was further increased after DOX-ss was linked by Au-S bonding, whereas the cytotoxicity of Au/DOX-ss LNRs was reduced after encapsulation of liposomes due to the biocompatibility of liposomes. Furthermore, the cell viability of OPC-Au/DOX-ss LNPs and Au/DOX-ss LNRs groups decreased by 41.86% and 34.64% compared with the Lipid DOX-ss group after NIR-ΙΙ irradiation.

Figure 5E,F show the IC_50_ values of polyphenol-conjugated gold nano-prodrugs in MCF-7/ADR cells after 24 h and 48 h incubation. The IC_50_ values of free DOX, Lipid DOX-ss, OPC-Au/DOX-ss LNPs, Au/DOX-ss LNRs, OPC-Au/DOX-ss LNPs + NIR-ΙΙ and Au/DOX-ss LNRs + NIR-ΙΙ in MCF-7/ADR were 36.41, 126.10, 3.29, 4.13, 3.03, and 3.91 μg/mL after 48 h, respectively. Compared with free DOX-ss, the IC_50_ of OPC-Au/DOX-ss LNPs and Au/DOX-ss LNRs were decreased by 91.68% and 89.26%, respectively. Compared with Lipid DOX-ss, the IC_50_ of OPC-Au/DOX-ss LNPs and Au/DOX-ss LNRs were decreased by 97.60% and 96.90%, respectively.

The above results indicated that polyphenol-conjugated gold nano-prodrugs OPC-Au/DOX-ss LNPs and DOX Au/DOX-ss LNRs could significantly improve the sensitivity for MCF-7/ADR cells, especially presented in the OPC-Au/DOX-ss LNPs group. It would provide a theoretical basis for synergistic photodynamic/photothermal therapy in the resistant treatment of breast cancer due to the reduction and stability of polyphenols in proanthocyanidins.

### 2.5. Intracellular Lysosomal Escape

**Figure 6:**
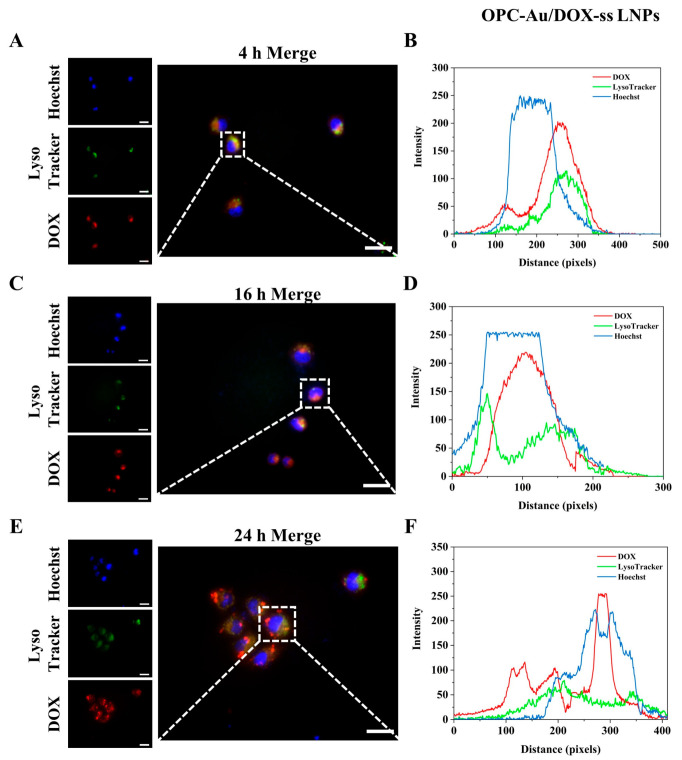
Lysosomal escape fluorescence imaging and co-localization of OPC-Au/DOX-ss LNPs. (**A**) The 4 h fluorescence imaging. (**B**) The 4 h fluorescence co-localization. (**C**) The 16 h fluorescence imaging. (**D**) The 16 h fluorescence co-localization. (**E**) The 24 h fluorescence imaging. (**F**) The 24 h fluorescence co-localization (The white dashed boxes show the lysosomal escape phenomenon, scale bar: 20 μm).

**Figure 7:**
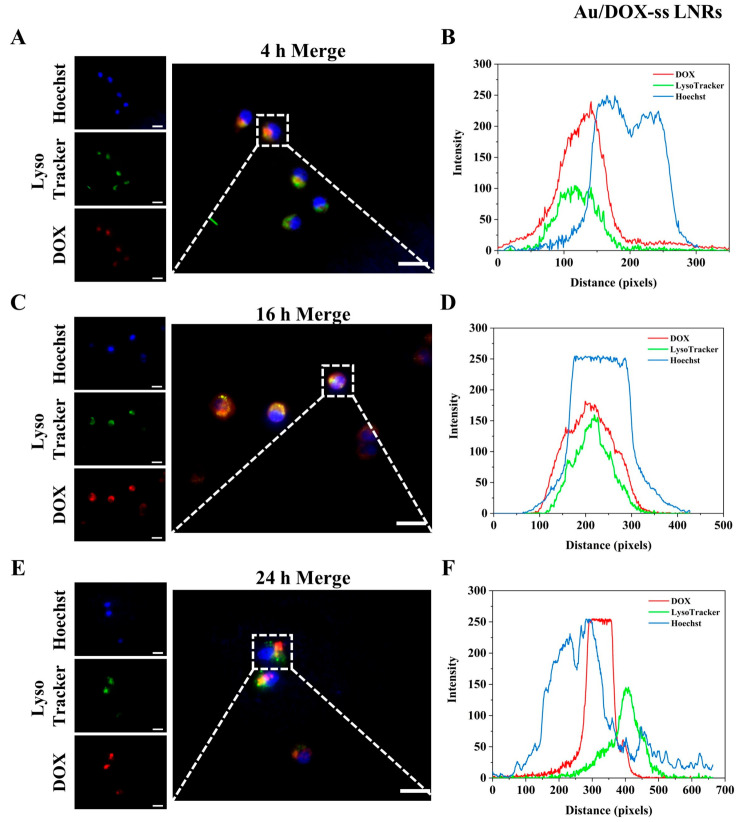
Lysosomal escape fluorescence imaging and co-localization of Au/DOX-ss LNRs. (**A**) The 4 h fluorescence imaging. (**B**) The 4 h fluorescence co-localization. (**C**) The 16 h fluorescence imaging. (**D**) The 16 h fluorescence co-localization. (**E**) The 24 h fluorescence imaging. (**F**) The 24 h fluorescence co-localization (The white dashed boxes show the lysosomal escape phenomenon, scale bar: 20 μm).

Nuclei and lysosomes were labeled with Hoechst 33342 and LysoTracker Green, respectively, to study lysosomal escape from OPC-Au/DOX-ss LNPs and Au/DOX-ss LNRs. The fluorescence intensity and localization of drug-loaded nanoparticles were analyzed by Image J. As shown in Figure 6 and Figure 7, after 4 h of co-incubation of MCF-7/ADR cells with OPC-Au/DOX-ss LNPs and Au/DOX-ss LNRs (Figure 6A,B and Figure 7A,B), the red fluorescence of DOX almost overlapped with the green fluorescence of LysoTracker, which indicated that the nanoparticles entered into the lysosome. As the incubation time was extended to 16 h (Figure 6C,D and Figure 7C,D), the red fluorescence separated from the green fluorescence and overlapped with the Hoechst blue fluorescence, indicating that the drug-loaded nanoparticles could escape from the lysosome into the cytoplasm and later into the nucleus. After 24 h incubation (Figure 6E,F and Figure 7E,F), the red fluorescence still overlapped with the blue fluorescence, but the overlapping part was reduced compared to 16 h, indicating that the drug-carrying nanoparticles were gradually released into the cytoplasm. The above results suggest that OPC-Au/DOX-ss LNPs and Au/DOX-ss LNRs are capable of lysosomal escape.

### 2.6. Intracellular ROS Detection

**Figure 8:**
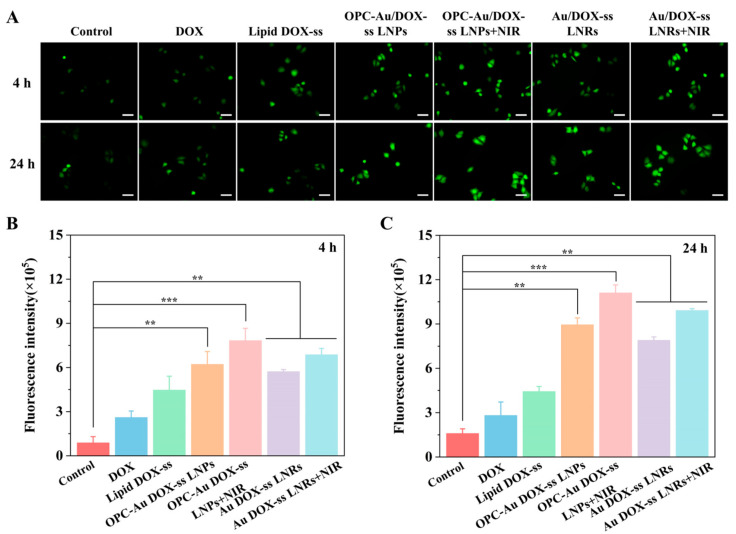
ROS detection in MCF-7/ADR cells induced by drug-loaded nanoparticles after 4 h and 24 h. (**A**) Fluorescent micrographs of ROS (scale bar: 20 μm). (**B**,**C**) Quantitative fluorescence intensity of ROS. ** and *** mean *p* < 0.01 and *p* < 0.001.

Figure 8 shows the ROS fluorescence graphs of each drug-loaded nanoparticle after 4 h and 24 h of incubation with MCF-7/ADR cells. The fluorescence of the free DOX group was weak, and the fluorescence intensity of ROS gradually rose with the increase in modification degree (Figure 8A). As shown in Figure 8B,C, the ROS fluorescence intensity of OPC-Au/DOX-ss LNPs and Au/DOX-ss LNRs after 24 h incubation was 5.65-fold and 4.99-fold higher than that of the control group. Interestingly, the ROS fluorescence intensity of OPC-Au/DOX-ss LNPs and Au/DOX-ss LNRs with NIR irradiation was further increased to 7.01-fold and 6.26-fold that of the control group, indicating that intracellular ROS levels were significantly increased due to the PTT effect, thereby inducing tumor apoptosis.

### 2.7. Effects of Polyphenol Structure on P-gp Inhibition in Multidrug-Resistant Breast Cancer Cells

**Figure 9:**
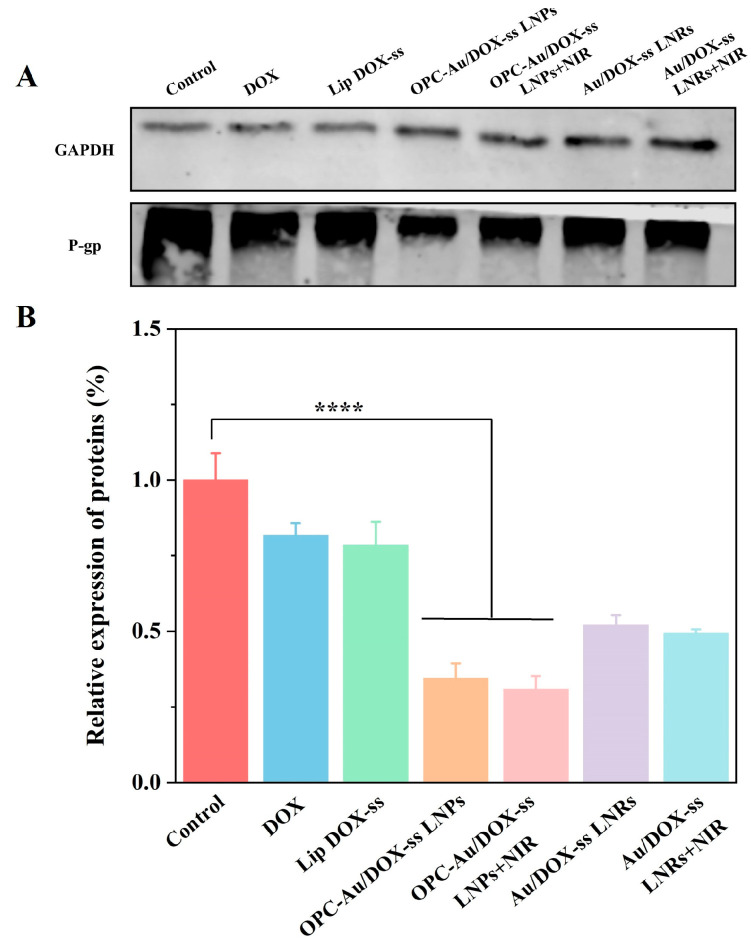
P-gp protein expression analysis. (**A**) Western blot analysis of P-gp. (**B**) Quantitative analysis of P-gp. **** means *p* < 0.0001.

The expression of P-glycoprotein (P-gp) in MCF-7/ADR cells was also examined by Western blot and quantified by Image Studio to investigate the changes in DOX resistance in MCF-7/ADR cells. The Western blot quantitative analysis (Figure 9) demonstrated that the relative expressions of P-gp protein in the OPC-Au/DOX-ss LNPs and DOX Au/DOX-ss LNRs group were reduced to 0.35 and 0.53 compared with the control group. Moreover, the relative expressions of P-gp protein in the OPC-Au/DOX-ss LNPs and DOX Au/DOX-ss LNRs group with NIR irradiation were reduced to 0.30 and 0.49 compared with the control group. The above results indicated that OPC-Au/DOX-ss LNPs could effectively inhibit the expression of P-gp protein due to the scavenging of free radicals of OPC, thus reducing the drug resistance of MCF-7/ADR cells and improving the antitumor effects.

## 3. Materials and Methods

### 3.1. Materials

Chloroauric acid trihydrate (HAuCl_4_⋅3H_2_O), hydroquinone, doxorubicin hydrochloride (DOX·HCl), proanthocyanidins (OPCs), hexadecyl trimethyl ammonium bromide (CTAB), hydrochloric acid (HCl), tetrahydrofuran dichloromethane, cholesterol, and reduced glutathione (GSH) were purchased from Shanghai Macklin Biochemical Co., Ltd. (Shanghai, China). Silver nitrate (AgNO_3_) and sodium borohydride (NaBH_4_) were purchased from American Merck Drugs & Biotechnology Co., Ltd. (Rahway, NJ, USA). 1,2-dioleoyl-sn-glycero-3-phosphoethanolamine-N-[poly(ethylene-glycol)]-hydroxy succinamide, PEG MW 2000 (DSPE-PEG 2000-NHS), 1,2-dioleoyl-sn-glycero-3-phosphoethanolamine-N-[sulfydryl(polyethylene glycol)-2000] (DSPE-PEG 2000-SH), and 1,2-dipalmitoyl-sn-glycero-3-phosphocholine (DPPC) were purchased from American Med. Chem. Express Biotechnology Co., Ltd. (Monmouth Junction, NJ, USA). LysoTracker Red and Hoechst 33342 were purchased from Solaibao Technology Co., Ltd. ( Beijing, China). All chemicals were analytical reagent grade and prepared without further disposal.

### 3.2. Synthesis of Au NRs

Gold nanorods (Au NRs) were prepared by the seedless growth method using hydroquinone as a reducing agent [22]. CTAB (10 mL, 100 mM) was added to a round-bottomed flask. Then, HAuCl_4_ (0.4 mL, 10 mM), AgNO_3_ (28 μL, 100 mM), HCl (30 μL, 1 M), and hydroquinone (0.54 mL, 100 mM) were added successively under vigorous stirring of 400 rpm at 25 °C for 15 min, the color changed from orange to light yellow and gradually clarifying and transparent. Afterward, ice-cold NaBH_4_ solution (10 μL, 0.1 mM) was added and mixed slowly for 15 s and stood at 25 °C for 24 h. The solution was centrifuged twice to remove CTAB at 12,000 rpm and 25 °C for 45 min. The Au NRs were dispersed in 1 mL deionized water and stored at 4 °C.

### 3.3. Synthesis of OPC-Au NPs

Gold nanoclusters (OPC-Au NPs) were prepared using proanthocyanidins as reducing agents [34]. HAuCl_4_⋅3H_2_O (30 μL, 0.24 mg/mL) and 24.97 mL water were mixed, stirred, and heated to boiling. Then, OPC (1 mL, 2 mg/mL, filtered with a 0.22 μm needle filter) was added rapidly, stirred, and heated for 10 min. The color of the solution changed from light yellow to dark red, which was obtained as OPC-Au NPs.

### 3.4. Synthesis of Lipid DOX-ss

DOX-ss was prepared based on previously described synthesis methods [43]. The intermediate product ethyl 2-(2-hydroxyethyl) disulfonyl methacrylate was obtained as follows. 2-hydroxyethyl disulfide (2.64 mL), triethylamine (4.20 mL), and tetrahydrofuran (100 mL) were added sequentially and maintained at 0 °C in an ice bath under N_2_ atmosphere. Subsequently, 50 mL of tetrahydrofuran solution containing methacryloyl chloride (2.04 mL) was added dropwise under vigorous stirring for 24 h at 20–25 °C. The product ethyl 2-(2-hydroxyethyl) disulfonyl methacrylate was obtained after filtration and rotary evaporation, dilution with ethyl acetate, and filtration.

Then, DOX·HCl (58 mg) and 4-dimethylaminopyridine (48 mg) were dissolved in dichloromethane (20 mL), and triphosgene (274 mg) was added and stirred for 30 min at room temperature under N_2_ protection. Subsequently, 2-(2-hydroxyethyl) disulfonyl methacrylate (20 μL) was added dropwise and stirred at room temperature for 24 h. DOX-ss was obtained after desiccation with anhydrous magnesium sulfate, filtration, concentration by rotary evaporation, and freeze-drying.

Lipid DOX-ss was synthesized by amidation reaction between DPSE-PEG2000-NHS and DOX with minor modifications [44]. DPSE-PEG2000-NHS (67 mg) and DOX-ss (20 mg) were dissolved in DMSO (10 mL), respectively. Then, triethylamine (12 μL) was added as a catalyst and stirred at 25 °C for 24 h. Lipid DOX-ss was obtained after dialysis and freeze-drying.

### 3.5. Synthesis of Polyphenol-Conjugated Gold NIR-ΙΙ Nano-Prodrugs

Thermosensitive liposomes OPC-Au/DOX-ss LNPs and Au/DOX-ss LNRs were synthesized based on previously described liposome synthesis methods with minor modifications [21,45].

Lipid DOX-ss (10 mg), dipalmitoyl phosphatidylcholine (25 mg), DSPE-PEG 2000-SH (18.5 mg) and cholesterol (12.5 mg) were dissolved in chloroform/methanol mixture (10 mL, volume ratio was 3:1). The liposome was treated after the solvent evaporation by rotary vacuum evaporation at 40 °C and vacuum drying at 40 °C for 24 h. Subsequently, the OPC-Au NPs solution (10 mL) was added to link with DSPE-PEG 2000-SH through Au-S affinity under vigorous stirring at 400 rpm at 25 °C for 24 h. The above procedure was centrifuged twice at 12,000 rpm for 15 min, and the precipitate was fixed to 1 mL to synthesis OPC-Au/DOX-ss LNPs.

Au/DOX-ss LNRs were synthesized according to the above-described method. OPC-Au NPs were only replaced by Au NRs and retained the other reaction conditions.

### 3.6. Particle Size and Zeta Potential Analysis

OPC-Au NPs, Au NRs, Lipid DOX-ss, OPC-Au LNPs, OPC-Au/DOX-ss LNPs, Au LNRs, and Au/DOX-ss LNRs were dissolved in ultrapure water, ultrasonically dispersed into a solution with a concentration of 500 μg/mL. The particle size and zeta potential were determined by nanoparticle sizer and zeta potential, respectively (*n* = 3).

### 3.7. UV–Vis Analysis

The changes in the UV absorption wavelength of each nanocarrier were analyzed by UV–visible absorption spectrophotometer (UV-2550 visible spectrophotometer, Shimadzu Corporation Ltd., Kyoto, Japan). The OPC-Au NPs and Au NRs were diluted to the same concentration with ultrapure water and dispersed uniformly by ultrasonication. After the pure water was zeroed, the UV absorption spectra of each sample at 400–1100 nm were determined.

### 3.8. FT-IR Analysis

The chemical structure and functional groups of each nanocarrier were analyzed using Fourier transform infrared spectroscopy (FT-IR, Nicolet iS20, Thermo Fisher Scientific Ltd., Waltham, MA, USA). OPC, OPC-Au NPs, DOX, DOX-ss, Lipid DOX-ss, OPC-Au/DOX-ss LNPs, and Au/DOX-ss LNRs were completely dried, mixed with potassium bromide, milled homogeneously to press the slices, and scanned in the 4000–400 cm^−1^ range was scanned for each sample.

### 3.9. Examination of Photothermal Conversion Performance

The photothermal conversion performance of different nanocarriers was investigated for four cycles under the 1064 nm NIR irradiation within 5 min at 1 W/cm^2^, respectively. The photothermal conversion efficiencies were calculated according to Equations (1)–(4).

(1) $$\theta \;=\;\frac{T-T_{0}}{T_{\max}-T_{0}}$$

*T* was the temperature of the solution at different moments in the natural cooling state, *T_max_* was the highest temperature of the solution that reached equilibrium under laser irradiation, and *T*_0_ was the initial temperature of the solutions.

Next, the curve was plotted with *t* and *−lnθ* as the y-axis and x-axis, respectively. The system heat transfer time constant (*τ_s_*) was calculated from the slope of the fitted straight line to determine the *hS*.

(2) $$\mathrm{hS}\;=\;\frac{\Sigma_{i}m_{i}C_{p,i}}{\tau_{s}}$$

*h* was the heat transfer coefficient, *S* was the surface area of the container, *m_i_* was the weight of components, and *C_p,i_* was the specific heat capacity of components.

Then, the energy released by absorption of light from an equal amount of pure aqueous solution (*Q*_0_) was calculated.

(3) $$Q_{0}=\frac{\mathrm{cm}\Delta T}{t}$$

*c* was the specific heat capacity of water, *m* was the weight of water, Δ*T* was the temperature drop of pure water solution after turning off the laser, and *t* was the heat dissipation time of pure water.

Finally, the photothermal conversion efficiency *ŋ* was obtained from the *hS* and *Q*_0_.

(4) $$ŋ\;=\;\frac{\mathrm{hS}(T_{\max}-T_{0})}{I(1-10^{-A})}$$

*A* was the absorbance of nanomaterials at 808 nm and 1064 nm, and *I* was the power of the laser.

### 3.10. In Vitro Drug Release

In vitro drug release of OPC-Au/DOX-ss LNPs and Au/DOX-ss LNRs was investigated under different pH and GSH conditions, respectively. A total of 3 mg of OPC-Au/DOX-ss LNPs and Au/DOX-ss LNRs were dispersed in PBS buffer solution (3 mL) corresponding to the conditions. The above liquids were put into dialysis bags with a molecular weight cut-off of 500 Da, respectively. The dialysis bags were then placed in the corresponding PBS solution (30 mL) and continuously shaken at 37 °C. The release solution (3 mL) was aspirated and supplemented with fresh PBS solution (3 mL) with corresponding conditions at regular intervals. The NIR group was irradiated with a 1064 nm laser for 5 min at regular intervals. The absorbance of DOX was measured at 480 nm using a UV–visible spectrophotometer (*n* = 5). The cumulative release rate was calculated according to Equation (5).

(5) $$Q_{t}\;(\%)=\frac{C_{n}V_{0}+V_{i}\sum_{i=1}^{n-1}C_{i}}{m}$$

*C_n_* was the concentration of the collected release fluid, V_0_ was the volume of PBS solution in the shaker, V_i_ was the sampling volume, *C_i_* was the drug concentration on the *i-th* displacement, and *m* was the total amount of nanoparticles.

### 3.11. Hemolytic Experiments

A total of 5 mL of mouse blood was centrifuged at 4 °C, 1000 rpm for 5 min, and then washed with saline to make the supernatant free of red color. Subsequently, RBC was obtained as a 2% cell suspension in saline for hemolytic analysis and stored at 4 °C. Saline (negative control, 0% hemolysis), 1% TritonX-100 (positive control, 100% hemolysis) and nanoparticles of different concentrations were added to RBC cell suspension (0.25 mL of 2% RBC suspension was transferred to 1.5 mL centrifugal tubes and 0.25 mL of nanoparticles were added). Next, samples were mixed and incubated at 37 °C in an incubator for 2 h and centrifuged at 3000 rpm for 5 min. Then, 100 μL of the supernatant was taken into a 96-well plate, and the absorbance was determined at 540 nm (*n* = 5). Finally, the hemolysis rate was calculated according to Equation (6).

*Hemolytic Rate* (%) = (*A_sample_* − *A_negative control_*)/(*A_positive control_* − *A_negative control_*) × 100% (6)

*A_sample_* was the absorbance of the experimental group at 540 nm, *A_negative control_* was the absorbance of the negative control group at 540 nm, and *A_positive control_* was the absorbance of the positive control group at 540 nm.

### 3.12. In Vitro Cytotoxicity

Human normal hepatocytes (L-02) and multidrug-resistant breast cancer cells (MCF-7/ADR) were inoculated in 96-well plates at a density of 10^4^/well for 24 h, respectively. Then, different concentrations of empty nanoparticles and drug-loaded nanoparticles were added to each well and incubated at 37 °C for 24 h and 48 h, respectively. Subsequently, MTT (100 μL) was added and incubated for 4 h. The absorbance of each well was measured at 490 nm (*n* = 5), and the cell survival rate was calculated according to Equation (7).

*Cell viability* (%) = (*A_sample_* − *A_control_*)/(*A_cell_* − *A_control_*) × 100% (7)

*A_sample_* was the absorbance of cells after incubation with nanomedicine at 490 nm, *A_control_* was the absorbance of the medium at 490 nm, and *A_cell_* was the absorbance of cells without treatment at 490 nm.

### 3.13. Lysosome Escape

To assess whether the nanocarriers entered the cell nucleus after lysosomal escape, MCF-7/ADR cells were inoculated in 6-well plates containing cell climbing slides and cultured for 12 h. Next, the drug-loaded nanoparticles were added and cultured for 4 h, 16 h, and 24 h after removing the old medium. Then, LysoTracker Red (Solaibao Technology Co., Ltd., Beijing, China) was added to fresh medium and incubated for 5 min. After removing the old medium, Hoechst 33342 (Solaibao Technology Co., Ltd., Beijing, China) was added to stain the nuclear cells for 15 min. Finally, the fluorescence labeled by the cells was tracked using a laser confocal microscope.

### 3.14. ROS Detection

MCF-7/ADR cells were inoculated in 6-well plates and cultured for 12 h. Next, the drug-loaded nanoparticles were added and cultured for 4 h and 24 h after removing the old medium. Then, cells were treated with the intracellular reactive oxygen indicator DCFH-DA in the dark at 37 °C for 20 min. Finally, the fluorescence intensity of ROS was observed by fluorescence microscopy after fixation with 4% cell tissue fixative for 20 min.

### 3.15. Expression of P-Glycoprotein (P-gp) Through Western Blot Examination

MCF-7/ADR cells were inoculated in 6-well plates with a density of 10^5^ cells per well and cultured for 12 h. Then, drug-loaded nanoparticles were added and cultured for 24 h after removing the old medium. The cells were collected and washed with pre-cooled PBS. The protein samples were collected after the cell lysis on ice for 15 min and the heat treatment by metal bath at 37 °C for 30 min. The above protein samples were separated by SDS-PAGE gel electrophoresis. Then, the protein bands were transferred from the gel to the PVDF membrane by wet transfer. After treatment with the primary antibody of the target protein, the unbound antibody was removed after rinsing, and the enzyme immunolocalization was performed after the treatment of the secondary antibody to the membranes. Finally, the gray values of protein were analyzed by development methods (*n* = 3).

### 3.16. Statistical Analysis

All data are presented as the mean ± standard deviation of five independent experiments. Statistical analysis between various groups was performed using GraphPad Prism software 8.0. *, **, ***, and **** were considered statistically significant and extremely statistically significant at *p* < 0.05, *p* < 0.01, *p* < 0.001, and *p* < 0.0001, respectively.

## 4. Conclusions

In conclusion, NIR-ΙΙ nano-prodrugs OPC-Au/DOX-ss LNPs and Au/DOX-ss LNRs were prepared and demonstrated excellent chem-photothermal therapeutic efficacy, pH-GSH-NIR-ΙΙ responsive release performance, good biocompatibility, and high selectivity toward MCF-7/ADR cells. The proanthocyanidins endowed the OPC-Au NPs with reductive characteristics and negatively charged surfaces for long-term circulation and targeted selectivity toward MCF-7/ADR cells. The OPC-Au/DOX-ss LNPs enabled the vector to possess photothermal and chemical abilities and exhibited mild chem-photothermal properties for in vivo treatment. The IC50 of OPC-Au/DOX-ss LNPs (3.03 μg/mL) combined NIR-ΙΙ irradation was significantly decreased by 97.60% compared with Lipid DOX-ss (126.10 μg/mL). In addition, it resulted in a significant inhibition of the expression of P-gp. Overall, OPC-Au/DOX-ss LNPs represented a promising photodynamic/photothermal therapy with multifunctional capabilities for drug-resistant treatment.

## Data Availability

The datasets used and/or analyzed during the current study are available from the corresponding author upon reasonable request.

## Supplementary Materials

The following supporting information can be downloaded at https://www.mdpi.com/article/10.3390/molecules30112334/s1, Figure S1: Effect of polyphenol structure on the growth of gold nanoparticles. Figure S2. Chemical analysis of polyphenol–gold nano-prodrugs. Figure S3: Selectivity analysis of polyphenol-conjugated gold nano-prodrugs toward L-02 and MCF-7/ADR cells after 24 h of incubation.
