# Supplementary material for: Proanthocyanidin-Conjugated NIR-ΙΙ Nano-Prodrugs for Reversing Drug Resistance in Photothermal Therapy
Source: Molecules. 2025 May 27;30(11):2334. doi: 10.3390/molecules30112334 (PMC12155667; doi:10.3390/molecules30112334)
Supplement: Supplementary file 1 [file molecules-30-02334-s001.zip › molecules-3641138-supplementary.pdf]

Proanthocyanidin-Conjugated NIR-II Nano-Prodrugs for Reversing Drug Resistance in Photothermal Therapy

Lan Cui [1,2,3], Weishuang Lou [1], Xin Wei [1], Mengdi Li [1], Mengyao Sun [1], Siyue Wang [1], Shuoye Yang [1], Lu Zhang [1], Guangzhou Zhou [1,*], Peng Li [2,*] and Lingbo Qu [3]

1 College of Biological Engineering, Henan University of Technology, Zhengzhou 450001, China; cuilanmm@haut.edu.cn (L.C.); 13598611016@163.com (W.L.); 18236139462@163.com (X.W.); 17513121786@163.com (M.L.); 17737879593@163.com (M.S.); w200408170546@163.com (S.W.); yangshuoyecpu@163.com (S.Y.); zhanglu@haut.edu.cn (L.Z.)
2 Institute for Complexity Science, Henan University of Technology, Zhengzhou 450001, China
3 College of Chemistry, Zhengzhou University, Zhengzhou 450001, China; qulingbo@zzu.edu.cn
* Correspondence: zhougz@haut.edu.cn (G.Z.); lipeng@haut.edu.cn (P.L.)

1.1. Effects of polyphenol structure on gold nanoparticles

As shown in Figure S1 A, the potential of Au NRs decreased from 31.4 mV to 16.3 mV, but the stability worsened as increasing the dose of $NaBH_4$ (HCl: 10 μL, Ag-NO_3: 23 μL).

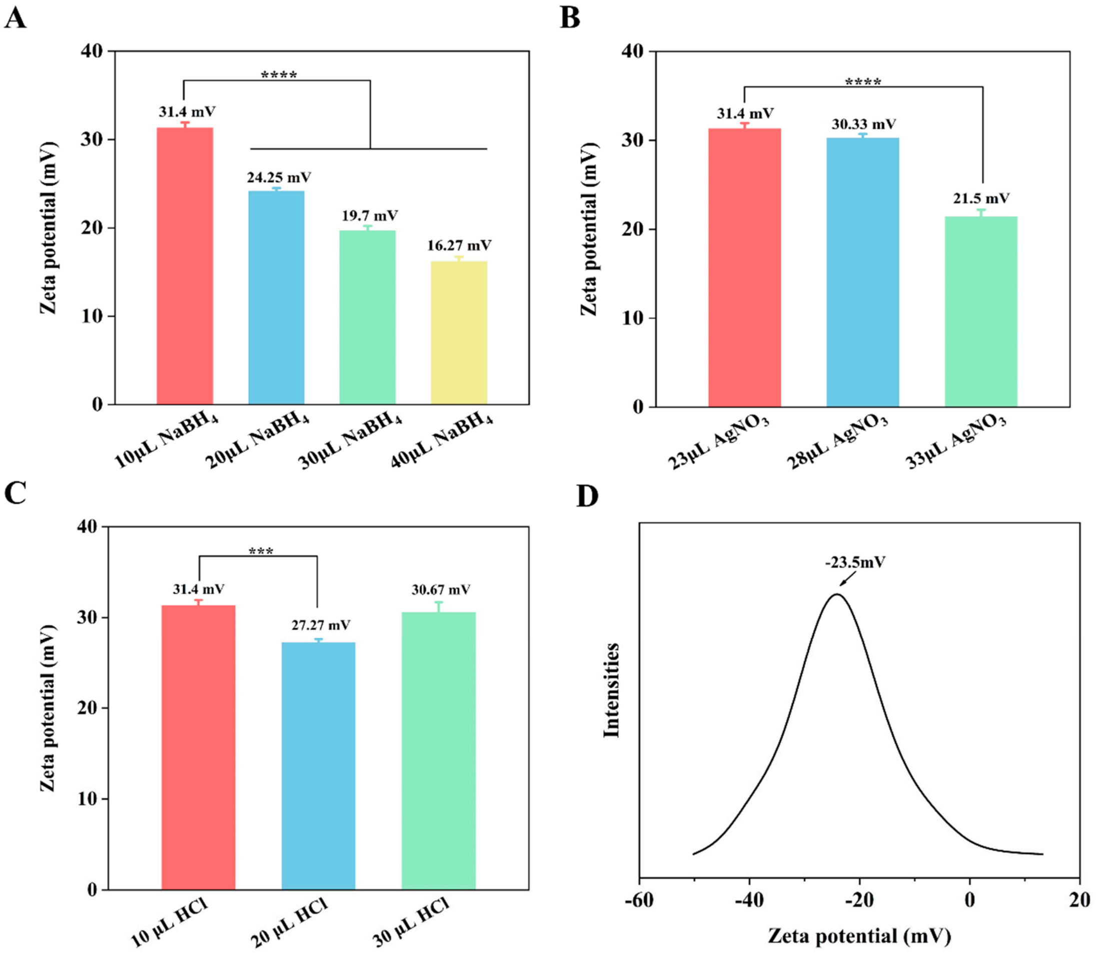

Figure S1. Effect of polyphenol structure on the growth of gold nanoparticles. (A) Effect of NaBH4 on the zeta potential of Au NRs. (B) Effect of AgNO3 on the zeta potential of Au NRs. (C) Effect of HCl on the zeta potential of Au NRs. (D) Potential analysis of OPC-Au NPs.

Figure S1 B revealed that the potential of Au NRs gradually reduced to 21.5 mV and became instable as increasing the dose of AgNO3 ($NaBH_4$: 10 μL, HCl: 10 μL). As shown in Figure S1 C, the potential of Au NRs also slightly declined to 30.67 mV with the increase of HCl dose ($NaBH_4$: 10 μL, $AgNO_3$: 23 μL).

As shown in Figure S1D, OPC-Au NPs exhibited excellent performance. The zeta potential was -23.5 mV. It has been found that positively charged carriers are easily captured by macrophages during blood circulation and cause inflammation and tissue damage upon binding with immune protein. However, negatively charged or uncharged nanomedicines perform excellent characteristics to achieve long circulation in vivo [26–28].

Therefore, OPC-Au NPs using proanthocyanidins as reducing and stabilizing agents through the one-pot method was more conducive in the subsequent study of drug-carrying nano systems.

1.2. Chemical characteristics of polyphenol conjugated gold nano-prodrugs

The FT-IR spectrums of OPC-Au/DOX-ss LNPs and Au/DOX-ss LNRs were shown in Figure S2 A-B. The broad and intense adsorption peak at 3392 cm-1 was corre-sponded to the stretching vibration peak of the hydroxyl on the aromatic ring of OPC. The peaks at 1614 cm-1, 1523 cm-1, and 1446 cm-1 were associated with the backbone vi-bration of the aromatic ring on the benzopyran ring. The peak shifts to 3430 cm-1 with a narrower peak shape compared with OPC, which illustrated the redox reaction be-tween the phenolic hydroxyl of proanthocyanidins and Au3+. The skeletal vibrational peaks of aromatic ring on the benzopyran ring of OPC-Au are shifted to 1635 cm-1, 1523 cm-1 and 1401 cm-1, which may be ascribed to the redox reaction of phenolic hydroxyl. The position and absorption peaks on the benzene ring framework were changed due to the enhanced electron donating ability of oxygen anion to the benzene ring [30,31]. These results suggested the successful synthesis of OPC-Au NPs.

In addition, the characteristic absorption peaks at 805 and 1220 cm-1 are associated with DOX, the C-OH and C-C characteristic peaks of DOX at 1285 and 1412 cm-1, the bi-carbonyl stretching vibration peak of DOX at 1614 cm-1 [32,33], and the appearance of S-S stretching vibration peak at 539 cm-1 proved that disulfide bond was successfully connected to DOX. 1739 cm-1 was the carbonyl stretching vibration peak of DSPE-PEG2000 [34], 2918 cm-1 and 2854 cm-1 represent the CH alkyl peaks of DSPE-PEG2000 [32], these results indicated that the liposomes successfully encapsu-lated DOX-ss. The characteristic absorption peak of Au-S bond at 765 cm-1 [35] indicat-ed that the OPC-Au NPs and Au NRs were attached to Lipid DOX-ss. OPC-Au/DOX-ss LNPs and Au/DOX-ss LNRs were successfully synthesized.

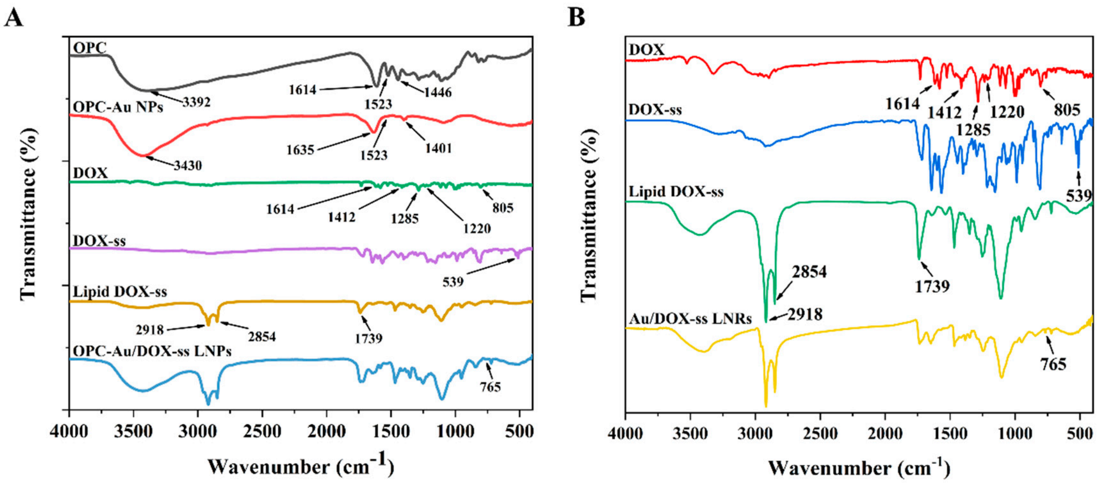

Figure S2. Chemical analysis of polyphenol-gold nano-prodrugs. (A) FT-IR of OPC-Au/DOX-ss LNPs. (B) FT-IR of Au/DOX-ss LNRs.

1.3. Targeting selectivity of polyphenol conjugated gold nano-prodrugs in multidrug-resistant breast cancer cells

As shown in Figure S3A-B, the cell viability OPC-Au NPs, Au NRs and lipid empty carriers were all above 80% after 24 h incubation in human normal hepatocytes (L-02) and multidrug-resistant breast cancer cells (MCF-7/ADR), showing good biosafety.

Figure S3C showed that DOX was more toxic to L-02 cells after 24 h of incubation (DOX: 2 μg/mL). The cell survival rates of OPC-Au/DOX-ss LNPs and Au/DOX-ss LNRs were both slightly declined compared with Lipid DOX-ss, but also above 80%, which were less toxic to normal cells, and exhibited a better biosafety.

However, polyphenol conjugated gold nano-prodrugs significantly improved the sensitivity of MCF-7/ADR cells, especially the sensitivity was significantly enhanced in OPC-Au/DOX-ss LNPs group than DOX Au/DOX-ss LNRs group (Figure S3D).

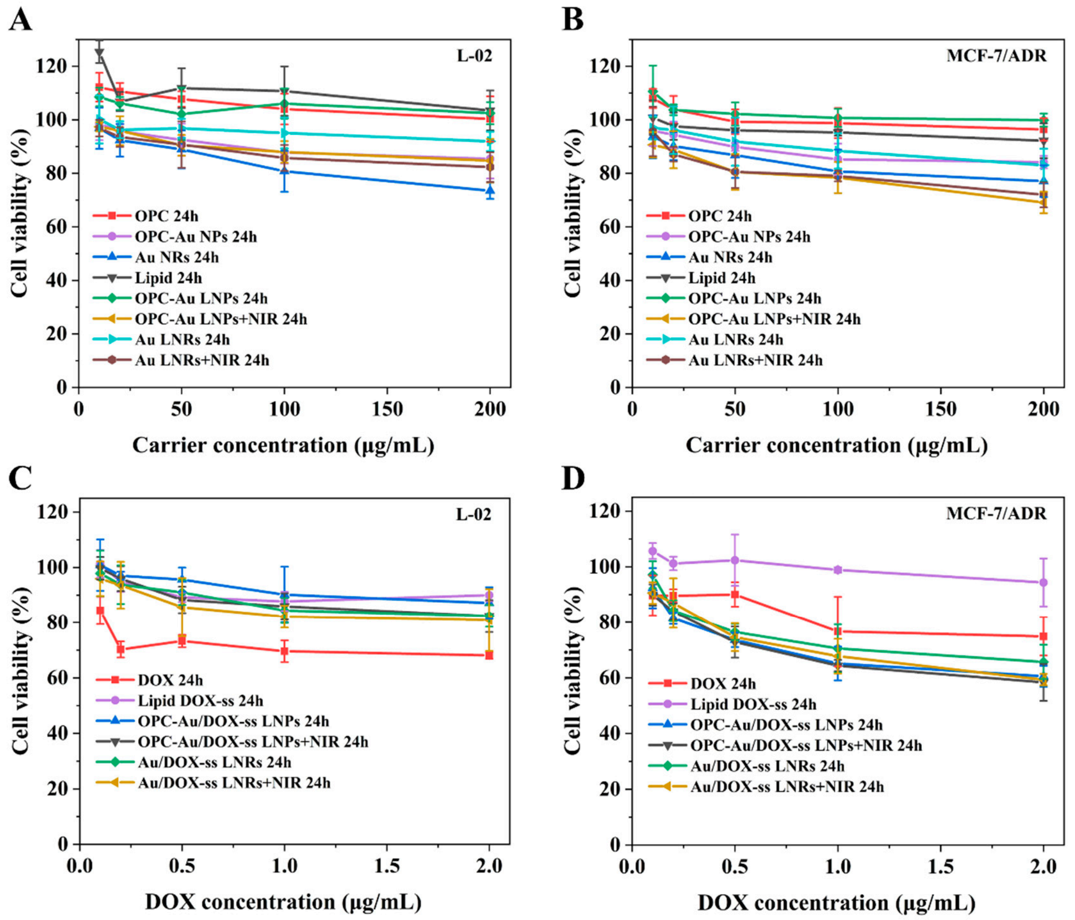

Figure S3. Selectivity analysis of polyphenol conjugated gold nano-prodrugs toward L-02 and MCF-7/ADR cells after 24 h of incubation. (A-B) Cell survival of empty carriers in L-02 and MCF-7/ADR cells. (C-D) Cell survival of polyphenol-gold nano prodrugs in L-02 and MCF-7/ADR cells.
